# Characterization of SpsQ from *Staphylococcus pseudintermedius* as an affinity chromatography ligand for canine therapeutic antibodies

**DOI:** 10.1371/journal.pone.0281171

**Published:** 2023-01-26

**Authors:** Hiroto Takeuchi, Chie Nakajima, Satoru Konnai, Naoya Maekawa, Tomohiro Okagawa, Masaru Usui, Yutaka Tamura, Yasuhiko Suzuki, Shiro Murata, Kazuhiko Ohashi

**Affiliations:** 1 Faculty of Veterinary Medicine, Department of Disease Control, Hokkaido University, Sapporo, Japan; 2 Faculty of Veterinary Medicine, Department of Advanced Pharmaceutics, Hokkaido University, Sapporo, Japan; 3 Division of Bioresources, International Institute for Zoonosis Control, Hokkaido University, Sapporo, Japan; 4 Institute for Vaccine Research and Development (HU-IVReD), Hokkaido University, Sapporo, Japan; 5 Department of Health and Environmental Sciences, School of Veterinary Medicine, Rakuno Gakuen University, Ebetsu, Japan; 6 Faculty of Veterinary Medicine, International Affairs Office, Hokkaido University, Sapporo, Japan; East China Normal University School of Life Sciences, CHINA

## Abstract

Coagulase-positive Staphylococci express protein A, which binds to host antibodies, to evade the immune system. Taking advantage of its specific binding to antibodies, protein A from *Staphylococcus aureus*, which is called SpA, is commonly used as an affinity chromatography ligand for human therapeutic antibodies. However, among four canine IgG subclasses (A, B, C, and D), only IgG-B binds to SpA strongly and establishing an efficient and robust purification scheme for canine therapeutic antibodies whose IgG subclass is A, C, or D remains difficult and depends on finding a suitable substitute to SpA. *S*. *pseudintermedius*, a major coagulase-positive Staphylococci found in dogs, expresses *spsQ* gene which is orthologous to *S*. *aureus spa*. We hypothesized that to serve *S*. *pseudintermedius* to better adapt to the dog immune system, SpsQ would bind to canine IgGs stronger than SpA, making it a better affinity chromatography ligand for canine therapeutic antibodies. To characterize SpsQ, we first determined the *spsQ* nucleotide sequence from *S*. *pseudintermedius* isolates. Based on the identified sequence, we prepared recombinant proteins containing the immunoglobulin-binding domains of SpA (r-SpA) and SpsQ (r-SpsQ) and determined their binding capacity for each canine IgG subclass. The binding capacity of r-SpsQ for IgG-B was almost as high as that of r-SpA. Interestingly, while both r-SpsQ and r-SpA showed no binding to IgG-C, the binding capacity of r-SpsQ for IgG-A and IgG-D was significantly higher than that of r-SpA. Finally, we performed affinity chromatography using r-SpsQ- or r-SpA-immobilized resin and revealed that the recovery rates of IgG-A and IgG-D using r-SpsQ were significantly higher than those using r-SpA. Our findings indicate that SpsQ has a strong potential to be used as an affinity chromatography ligand for canine therapeutic antibodies of subclass A, B, and D.

## Introduction

Coagulase-positive staphylococci (CoPS) are commensal bacteria in humans and species of our companion and livestock animals. CoPS express various molecules, including protein A, that help them evade the host immune system [1,2]. Protein A is a cell wall-anchored or secretory protein that binds to the Fc and Fab regions of immunoglobulins (Igs) [3,4]. Among the CoPS, *Staphylococcus aureus* is commonly isolated from human skin and mucosal areas [5,6]. Protein A from *S*. *aureus* is called SpA, which is encoded by the *spa* gene. A study in mice showed that a SpA mutant *S*. *aureus*, whose SpA cannot bind to Igs, was killed efficiently by the host immune cells, and the infected mice survived after the challenge with a lethal dose of wild-type *S*. *aureus* [7], demonstrating that the Ig-binding ability of SpA is a critical factor for the evasion of the host immune system.

Taking advantage of the strong and specific binding of SpA to human IgGs, SpA is commonly used for the purification of human therapeutic antibodies. Canine IgGs are classified into four subclasses, IgG-A, IgG-B, IgG-C, and IgG-D, that correspond to human IgG2, IgG1, IgG3, and IgG4, respectively [8,9]. The biological functions, including antibody-dependent cell-mediated cytotoxicity and complement-dependent cytotoxicity, are dependent on canine IgG subclasses. Because the requirements of those two functions for a therapeutic antibody are dependent on the functions and distribution of the target molecule, a therapeutic antibody may need to incorporate every canine IgG subclass. However, among the four canine IgG subclasses, only IgG-B is reported to be strongly bound to SpA, and IgG-A, IgG-C, and IgG-D show no or little binding to SpA [9]. Thus, using SpA as an affinity chromatography ligand is inefficient for the purification of IgG-A, IgG-C, and IgG-D, and an efficient and robust purification scheme for canine therapeutic antibodies remains to be established.

To improve the purification efficiency for canine IgGs, we sought to identify an appropriate binding molecule, which could bind to canine IgGs more strongly than SpA. We took our clue from the known difference in the major CoPS between humans and dogs: *S*. *pseudintermedius* is often found on canine skin and mucosal areas [10–13]. The *S*. *pseudintermedius* gene orthologous to *spa* is *spsQ* which encodes SpsQ [14]. A previous study showed that SpsQ is expressed on the cell wall of *S*. *pseudintermedius* and binds to the Fc region of canine IgGs [15,16]. Although some strains lack *spsQ* gene, it is widely detected as a lineage-associated gene among *S*. *pseudintermedius* isolates [12,17,18].

We hypothesized that SpsQ may bind to canine IgGs more strongly than SpA, because that would serve *S*. *pseudintermedius* in its adaption to the dog immune system. If this was the case, SpsQ could be used as a potentially superior affinity chromatography ligand for canine antibodies. However, the characteristics of SpsQ binding to each canine IgG subclass have not been reported and the utility of SpsQ for the purification of canine antibodies remains to be elucidated. In this study, we addressed both issues. To evaluate the binding capacity of SpsQ for each canine IgG subclass and compare them to those of SpA, we first determined the nucleotide sequence of *spsQ* from *S*. *pseudintermedius* isolates. Next, we prepared histidine-tagged recombinant proteins containing the Ig-binding domains (Ig-BDs) of SpsQ (r-SpsQ) and SpA (r-SpA), and determined the binding capacity of r-SpsQ or r-SpA for each canine IgG subclass. Finally, r-SpsQ or r-SpA was immobilized onto the affinity chromatography resin, and the purification efficiency for each canine IgG subclass using r-SpsQ or r-SpA was determined and compared.

## Material and methods

### Determination of the *spsQ* gene sequence

The genomic DNA of *S*. *pseudintermedius* or *S*. *aureus* was extracted from bacterial cultures using a QIAamp DNA Mini Kit (Qiagen, Tokyo, Japan) or an InstaGene matrix (Bio-Rad, Hercules, CA, USA) [13]. The open reading frame (ORF) of *spsQ* gene was amplified from *S*. *pseudintermedius* genomic DNA by PCR using TaKaRa Ex Taq polymerase (Takara Bio, Tokyo, Japan) with the primers 5′-ACC GCT CTA TTT TTA GGT TAA TCA AA-3′ and 5′-ACT GTG ACG TAA CAA CTC AAT GC-3′, which were designed based on the complete genome sequence of *S*. *pseudintermedius* ED99 registered in the GenBank database (CP002478.1). The following cycling conditions were performed: initial denaturation at 96°C for 2 minutes, 37 cycles of denaturation at 98°C for 10 seconds, annealing at 55°C for 5 seconds, and extension at 72°C for 2 minutes. The amplicon was purified using the FastGene gel/PCR extraction kit (Nippon Genetics, Tokyo, Japan), and sequenced (Eurofins Genomics, Tokyo, Japan). The ORF sequence thus determined was translated into the deduced amino acid sequences using Mega software (version 7) and aligned using the MUSCLE method [19,20].

### Expression and purification of r-SpsQ and r-SpA

The Ig-BDs of SpsQ or SpA were amplified by PCR using KOD One PCR Master Mix -Blue- (TOYOBO, Osaka, Japan) with the primers with restriction sites for *Nde*I and *Xho*I (underlined) (SpsQ: 5′-AAG CAT ATG AAC GCA GAA GCG CAA CAA AAC-3′ and 5′-ATC CTC GAG TTA TTT AGG CGC TTG GCT GTC ATT-3′; SpA: 5′-AAG CAT ATG CAC GAT GAA GCT CAA CAA AAT GC-3′; and 5′-ATC CTC GAG TTA TTT TGG TGC TTG AGC ATC GTT-3′). The following cycling conditions were performed: 35 cycles of denaturation at 98°C for 10 seconds, annealing and extension at 68°C for 5 seconds. The amplicon was purified using the FastGene gel/PCR extraction kit (Nippon Genetics), digested with *Nde*I and *Xho*I (New England BioLabs, Ipswich, MA, USA), and cloned into the expression vector pET-19b with N-terminal his-tag sequence (Merck, Darmstadt, Germany). Rosetta–gami B (DE3) competent cells (Merck) were transformed with the constructed expression vector by electroporation using the Electroporation System Gene Pulser Xcell (Bio-Rad).

To express recombinant proteins containing the Ig-BDs of SpsQ (r-SpsQ) or SpA (r-SpA), a single colony of the transformed cells was cultured in LB broth containing 50 μg/ml Carbenicillin (Sigma-Aldrich, St. Louis, MO, USA) at 37°C. When an OD600 of 0.6 was reached, 1 mM Isopropyl β-D-1-thiogalactopyranoside (IPTG; Protein Ark, Sheffield, United Kingdom) was added, and culturing continued for another four hours. Bacterial cells were collected by centrifugation at 10,000 × g for 10 min. The pelleted bacteria were lysed and incubated at room temperature (RT) for 30 min with Bugbuster Protein Extraction Reagent (Millipore, Burlington, MA, USA) containing 1 tablet/50 ml complete EDTA-free Protease Inhibitor Cocktail (Roche; Basel, Switzerland), 5 U/ml rLysozyme Solution (Millipore), and 1,000 U/ml Benzonase Nuclease (Millipore). After centrifugation at 10,000 × g for 10 min, recombinant protein was purified from the supernatant using Ni Sepharose 6 Fast Flow (Cytiva, Uppsala, Sweden). The buffer was replaced with phosphate-buffered saline (PBS; FUJIFILM Wako Pure Chemical) using Amicon Ultra-4 10 kDa filter (Merck). Protein concentration was measured using a NanoDrop8000 spectrophotometer (Thermo Fisher Scientific, Waltham, MA, USA). Sodium dodecyl sulfate-polyacrylamide gel electrophoresis (SDS-PAGE) was performed using 2× Laemmli Sample Buffer (Bio-Rad) containing 2-mercaptoethanol in reducing conditions. The sample was incubated at 96°C for 5 min and separated by electrophoresis using SuperSep Ace 5%–20% gel (FUJIFILM Wako Pure Chemical). Precision Plus Protein All Blue Standards (Bio-Rad) was used as a protein standard. The gel was stained using the Quick-CBB kit (FUJIFILM Wako Pure Chemical). The purities of recombinant proteins were evaluated by densitometry using CS Analyzer version 3.0 software (Atto, Tokyo, Japan).

### Preparation of chimeric monoclonal IgGs

The preparation of a rat-dog chimeric anti-PD-L1 monoclonal antibody (c4G12) with IgG-D heavy chain constant region was reported previously [21]. To prepare chimeric antibodies with IgG subclasses IgG-A, IgG-B, or IgG-C, the same rat heavy chain variable region was fused to the constant region of dog IgG-A (GenBank accession number AF354264), IgG-B (AF354265), or IgG-C (AF354266), respectively. The nucleotide sequences of the chimeric antibody light chain and heavy chain were inserted into the pDC62c5-U533 expression vector [22] and the expression plasmid was extracted and purified using a NucleoBond Xtra Midi Kit (Takara Bio). The purified plasmid was then transfected into Chinese hamster ovary DG44 cells (Thermo Fisher Scientific) using Lipofectamine LTX reagent (Thermo Fisher Scientific). Transfectants were cloned by limiting dilution in CD OptiCHO medium supplemented with 4 mM GlutaMAX-I (Thermo Fisher Scientific) to obtain stable producer cell lines. The resultant cell clones that expressed chimeric antibodies with each IgG subclass were cultured in OptiCHO medium or Dynamis medium (Thermo Fisher Scientific) supplemented with 4 mM GlutaMAX-I (Thermo Fisher Scientific) for 14 days on an orbital shaker operated at 125 rpm. For cultures in Dynamis medium, 4 g/l, 4 g/l, and 6 g/l glucose (Fujifilm Wako Pure Chemical) was added on days 3, 5, and 7, respectively, and 3.3% (v/v) EfficientFeed B+ (Thermo Fisher Scientific) was added on days 3, 5, 7, and 10. Cell culture supernatant was harvested on day 14 and subjected to antibody purification using Ab-Capcher ExTra (Protenova, Kagawa, Japan) or Protein G Sepharose 4 Fast Flow (Cytiva). The buffer was replaced with PBS (FUJIFILM Wako Pure Chemical) using PD MidiTrap G25 (Cytiva) and the proteins were concentrated using Amicon Ultra-0.5 10 kDa (Merck). The concentration of chimeric antibodies was measured using a NanoDrop 8000 Spectrophotometer (Thermo Fisher Scientific).

### Binding of SpsQ and SpA with canine IgGs

The binding capacity of r-SpsQ or r-SpA for canine IgGs was evaluated by ELISA. The recombinant protein was biotinylated using the Biotinylation Kit/Biotin Conjugation Kit (Fast, Type A)—Lightning-Link (Abcam, Cambridge, UK). A 96-well ELISA microplate (Nunc maxisorp; Thermo Fisher Scientific) was coated with 1 μg/ml of each canine IgG subclass diluted in PBS at RT for 1 h. After washing with PBS containing 0.05% Tween20 (Kanto Chemical, Tokyo, Japan), the plate was blocked with SuperBlock T20 (PBS) Blocking Buffer (Thermo Fisher Scientific) at 37°C for 1 h. Biotinylated r-SpsQ or r-SpA was added to the plate at various concentrations (from 2 ng/ml to 0.5 ng/ml in 2-fold dilution series), followed by incubation at RT for 1 h. The reaction was developed using horseradish peroxidase (HRP)-conjugated NeutrAvidin protein (Thermo Fisher Scientific) and TMB One Component Substrate (Bethyl Laboratories, Montgomery, TX, USA) and stopped with 0.18 M H_2_SO_4_. SuperBlock T20 (PBS) Blocking Buffer (Thermo Fisher Scientific) was used as a dilution buffer for recombinant proteins and NeutrAvidin. The absorbance was measured at 450 nm using the MTP-900Lab multiplate reader (CORONA ELECTRIC, Ibaraki, Japan).

### Purification of canine IgGs from the cell culture supernatant using r-SpsQ or r-SpA

To assess the applicability of r-SpsQ for affinity chromatography, we prepared chromatography resins immobilized with r-SpsQ or r-SpA. For the preparation of coupling solutions, r-SpsQ or r-SpA was diluted in 0.2 M NaHCO_3_ buffer, containing 0.5 M NaCl, pH 8.3 at the concentration of 10 mg/ml. The coupling solution was mixed with the half volume of NHS-activated Sepharose 4 Fast Flow resin (Cytiva). Following the incubation at 4°C overnight, the resins are blocked with 0.1 M Tris-HCl buffer, pH 8.5 at RT for a few hours. The resins were washed with 3 × bed volume of 0.1 M Tris-HCl buffer, pH8.5, followed by the wash with 0.1 M acetate buffer, containing 0.5 M NaCl, pH 4.0. This wash cycle was repeated six times. The prepared resin was preserved in 20% ethanol (Kanto Chemical) solution. The cell culture supernatant containing canine IgG-A, IgG-B, and IgG-D was prepared as described above. Resins with immobilized r-SpsQ or SpA were packed in a 1.2-ml Bio-Spin chromatography column (Bio-Rad) with a bed volume of 250 μl and equilibrated twice with 1 ml (4 × bed volume) of PBS (FUJIFILM Wako Pure Chemical). Then 1 ml of cell culture supernatant was loaded onto the column, and the resins were washed with 1 ml (4 × bed volume) of PBS (FUJIFILM Wako Pure Chemical). Antibodies were eluted from the resins by adding 1 ml of Glycine-HCl, pH 2.0 (Cytiva). The eluted sample was neutralized with Tris-HCl buffer, pH 9.0. All steps were performed in gravity flow.

### Measurement of canine IgGs concentration

A 96-well ELISA microplate (Nunc maxisorp, Thermo Fisher Scientific) was coated with 10 μg/ml of dog IgG1 antibody (A40-120A, Bethyl Laboratories) targeting canine IgG-A and IgG-D or dog IgG2 antibody (A40-121A, Bethyl Laboratories) targeting canine IgG-B, both diluted in PBS at RT for 1 h. After washing with PBS containing 0.05% Tween20 (Kanto Chemical), the plate was blocked with PBS containing 0.05% Tween20 (Kanto Chemical) and 1% bovine serum albumin (BSA) (Thermo Fisher Scientific) (1% BSA-PBS-T) at 37°C for 1 h. Samples were prepared at 1:2,000 dilution, and added to the plate, followed by incubation at RT for 1 h. The reaction was developed using HRP-conjugated dog IgG1 antibody (A40-120P, Bethyl Laboratories) or HRP-conjugated dog IgG2 (A40-121P, Bethyl Laboratories) and TMB One Component Substrate (Bethyl Laboratories) and then stopped with 0.18 M H_2_SO_4_. We used 1% BSA-PBS-T as a dilution buffer for the collected samples and the detection antibody. The absorbance was measured at 450 nm with the MTP-900Lab multiplate reader (CORONA ELECTRIC).

### Statistical analysis

Tukey’s test was used for multiple comparisons. A two-tailed t-test was used for the comparison of two groups. A *p*-value of less than 0.05 was considered statistically significant. JMP 16 (SAS Institute, Cary, NC, USA) was used for all statistical analyses.

## Results

### Determination of the nucleotide sequences of s*psQ* from *S*. *pseudintermedius* isolates

**Fig 1:**
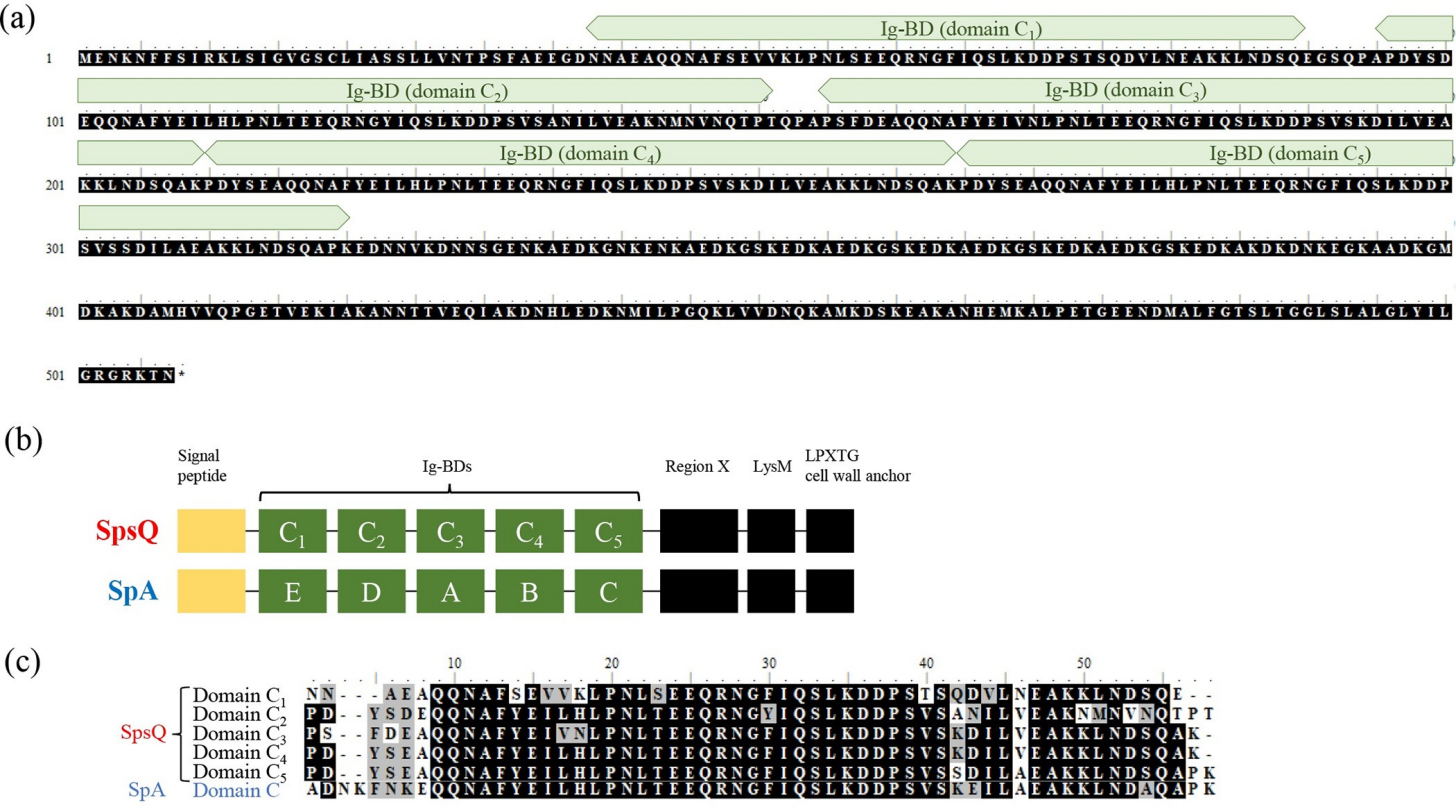
Determination of the Nucleotide Sequence of *spsQ* from *S*. *pseudintermedius* Isolates. (a) Alignment of SpsQ. Predicted Ig-BDs are indicated. (b) Schematics of the predicted functional motifs of SpsQ and SpA. (c) Alignment of each Ig-BD from SpsQ with domain C from SpA of *Staphylococcus aureus* subsp. aureus NCTC 8325 (GenBank accession number; CP000253.1).

To determine the nucleotide sequences of *spsQ*, the ORF sequence was amplified from the genomic DNA of *S*. *pseudintermedius* isolates. The ORF nucleotide sequence of *spsQ* was 1,569 bp in length. The prediction of functional domains using InterProScan revealed that SpsQ has five Ig-BDs (domain C_1–5_) (Fig 1A). In comparison, the Ig-BDs of SpA have been defined into functional units E, D, A, B, and C (Fig 1B) [23–25]. However, all five Ig-BDs of SpsQ showed high sequence similarities to each other and correspond to domain C of SpA of *S*. *aureus* subsp. aureus NCTC 8325 (Fig 1C).

### Preparation of recombinant proteins containing the immunoglobulin-binding domains from SpsQ and SpA

**Fig 2:**
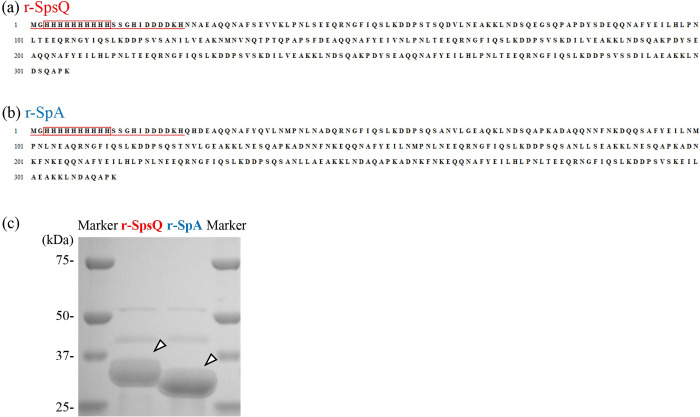
Preparation of recombinant SpsQ and SpA. The amino acid sequences of (a) recombinant SpsQ (r-SpsQ) and (b) recombinant SpA (r-SpA). Amino acid residues derived from the expression vector are underlined. The N-terminal 10 × his-tag sequence is shown by a red box. (c) SDS-PAGE of the purified recombinant proteins. Arrowheads indicate the specific protein bands with an expected molecular weight under reducing conditions.

To assess the IgG binding capacity of SpsQ or SpA for each canine IgG subclass, we prepared the recombinant proteins containing the binding domains of SpsQ (r-SpsQ) and SpA (r-SpA). The Ig-BDs were expressed with N-terminal his-tag and purified (Fig 2A and 2B). The recombinant proteins, r-SpsQ and r-SpA, were analyzed by SDS-PAGE for their purities. The purities of r-SpsQ and r-SpA were >90%. The molecular weights of r-SpsQ and r-SpA were estimated at approximately 35 kDa in reducing conditions (Fig 2C), consistent with the theoretical molecular weights of 34.6 kDa and 35.5 kDa, respectively, calculated from the deduced amino acid sequences.

### Binding capacity of r-SpsQ or r-SpA to canine IgGs

**Fig 3:**
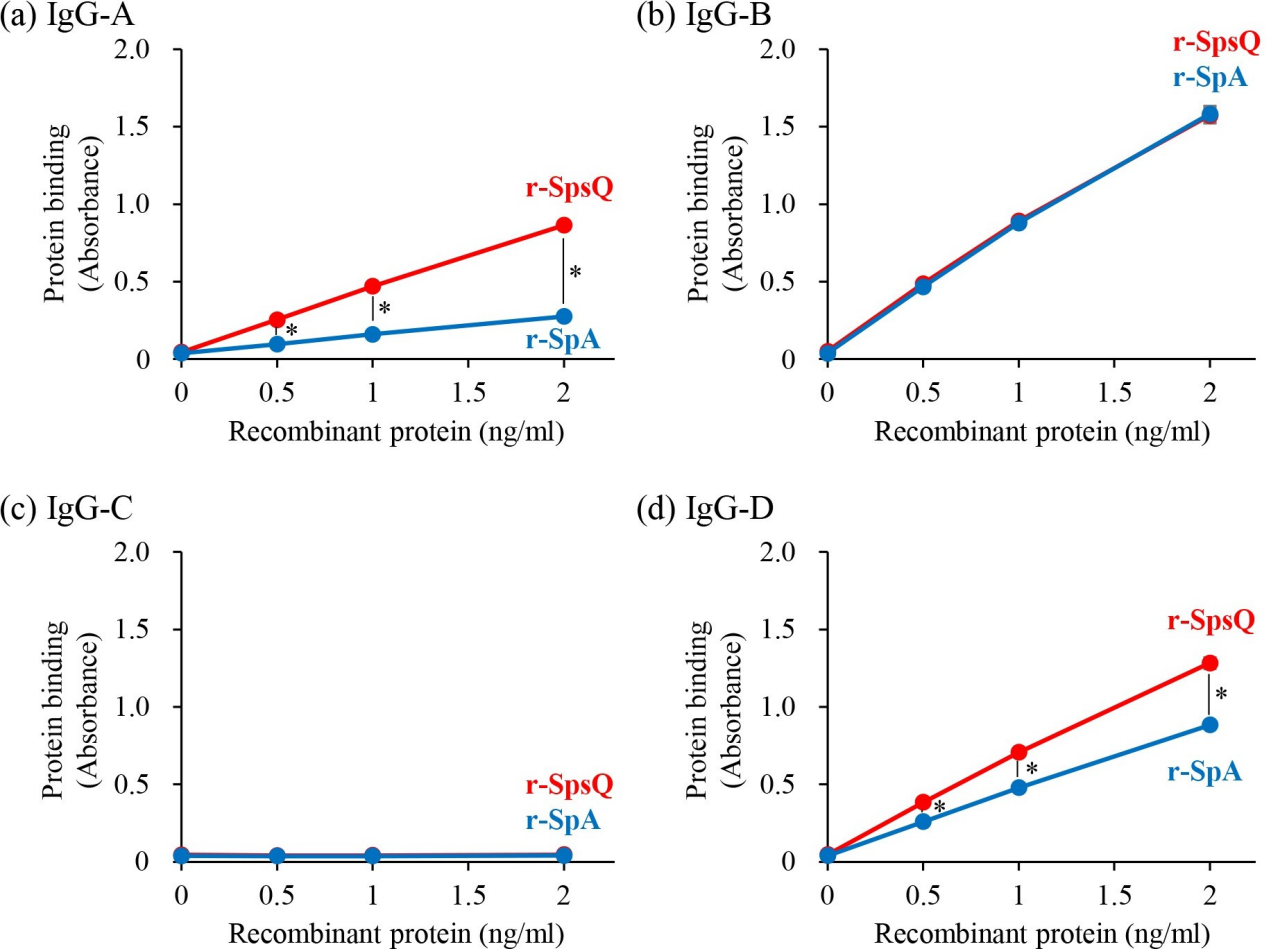
Evaluation of the binding of r-SpsQ or r-SpA to Canine IgGs. The comparison of the binding of r-SpsQ and r-SpA to canine (a) IgG-A, (b) IgG-B, (c) IgG-C, and (d) IgG-D, evaluated by ELISA. Briefly, canine IgGs were coated on the ELISA plates and biotinylated r-SpsQ or r-SpA was added onto the plate at various concentrations (horizontal axis). The binding of r-SpsQ or r-SpA was detected with HRP-conjugated Avidin and colorimetric substrate. The y-coordinate of each dot represents the average absorbance obtained from three independent experiments. Error bars represent the standard deviation. Tukey’s test was used for statistical comparison (*: *p* < 0.01).

Next, four types of canine chimeric antibodies with different constant regions (from IgG-A, IgG-B, IgG-C, or IgG-D) were prepared and the IgG binding capacity of r-SpsQ or r-SpA for each IgG subclass was evaluated by ELISA. The binding capacity of r-SpsQ for IgG-B was relatively high and statistically indistinguishable from that of r-SpA. Also, both r-SpsQ and r-SpA showed no binding to IgG-C. However, the binding capacity of r-SpsQ for both IgG-A and IgG-D was significantly higher than that of r-SpA at every IgG concentration (Fig 3A–3D).

### Purification of canine IgGs using r-SpsQ- or SpA-immobilized resins

**Fig 4:**
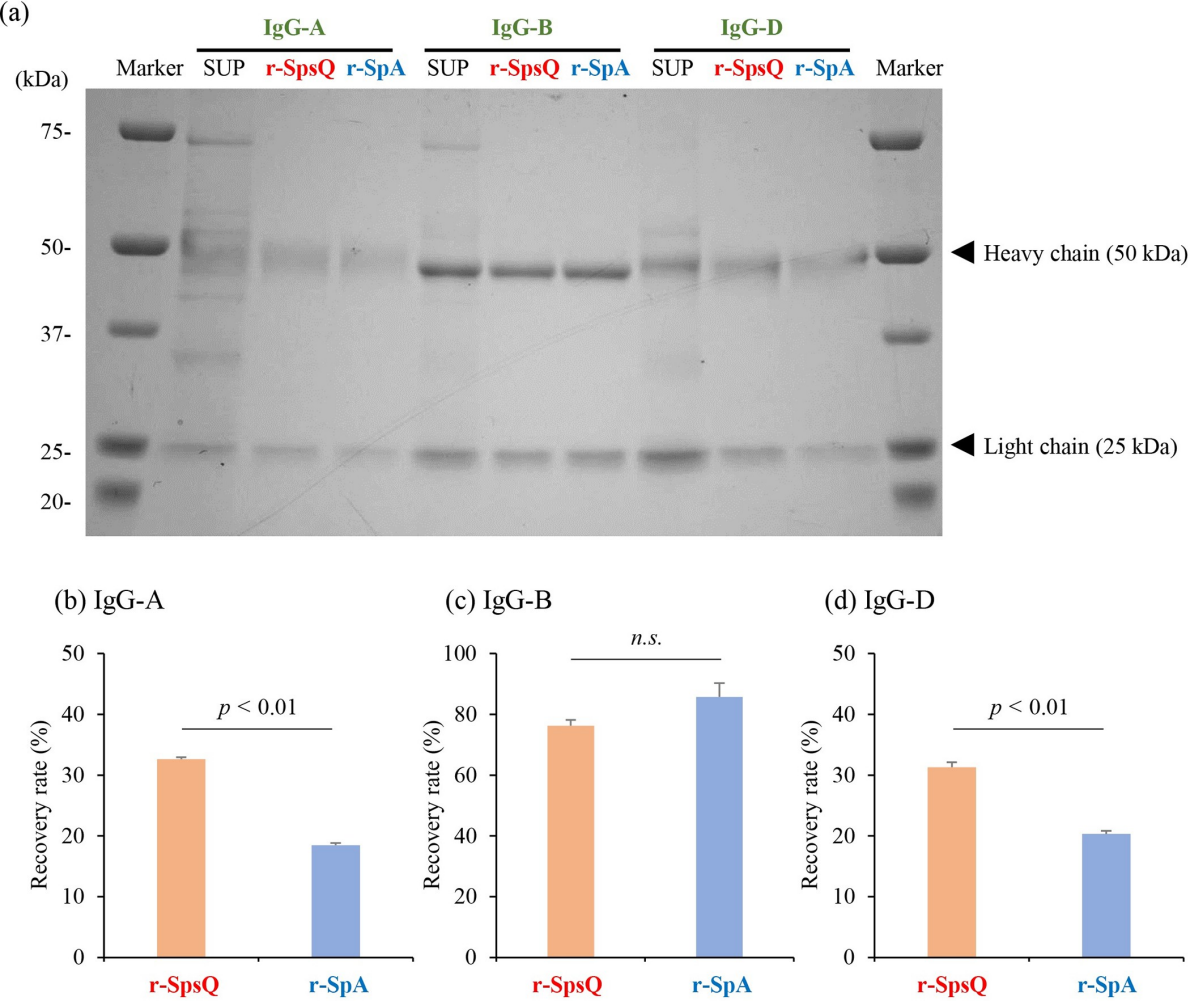
Purification of canine IgGs using r-SpsQ and r-SpA. (a) SDS-PAGE of the cell culture supernatant (SUP) and the recovered canine IgG-A, IgG-B, and IgG-D using r-SpsQ- or r-SpA-immobilized resin under reducing conditions. The recovery rate of (b) IgG-A, (c) IgG-B, and (d) IgG-D using r-SpsQ- or r-SpA-immobilized resin. The recovery rate is expressed as the recovered fraction (percentage) of the total amount of antibody loaded onto the resins (recovery rate % = amount (μg) of recovered antibody/loaded antibody ×100). Data points represent the average of three independent experiments. Error bars represent the standard deviation estimated from triplicates. A two-tailed t-test was used to compare groups.

Based on the results of the binding assay, we hypothesized that the purification efficiency of canine IgGs could be improved by using SpsQ as an affinity chromatography ligand. To compare the potential utility of SpsQ and SpA as affinity chromatography ligands, we established affinity chromatography for canine IgGs using 250 μl (bed volume) of resin immobilized with r-SpsQ or r-SpA. A cell culture supernatant containing canine IgG-A, IgG-B, or IgG-D was prepared at a concentration of approximately 0.7 g/L. Canine IgG-C was excluded from the purification test because both r-SpsQ and r-SpA did not bind to IgG-C at all (Fig 3C). Recovered canine IgGs were analyzed with SDS-PAGE for purity. We observed the removal of impurities from the cell culture supernatant by using each resin (Fig 4A). To assess the purification efficiency, the recovery rate was calculated as the recovered fraction of the total amount of antibody loaded onto the resins. The recovery rate of IgG-B using r-SpsQ- or r-SpA-immobilized resin was as high as approximately 80% with no statistically significant difference. The recovery rate of IgG-A using r-SpsQ-immobilized resin (33%) was 1.7-fold higher than that using r-SpA-immobilized resin (19%, *p* < 0.01). Similarly, the recovery rate of IgG-D using r-SpsQ-immobilized resin (31%) was 1.5-fold higher than that using r-SpA-immobilized resin (20%, *p* < 0.01) (Fig 4B–4D).

## Discussion

Therapeutic antibodies have been widely used and have become an essential pharmaceutical modality in human medicine because antibody drugs have high target specificity and long half-life in blood compared to traditional small-molecule drugs. The application of therapeutic antibodies has recently been expanded to companion animals. There are some effective therapeutic antibodies for dogs that are commercially available or under clinical pilot studies. Previously, our laboratory developed an immune checkpoint inhibitor, a rat-dog chimeric anti-PD-L1 monoclonal antibody (c4G12), for canine cancer treatment and conducted a clinical pilot study using c4G12 in dogs with pulmonary metastatic oral malignant melanoma (OMM). Treatment with c4G12 prolonged the survival of dogs compared to standard therapies (historical comparison) and induced tumor response in some dogs [26]. In addition to our therapeutic antibody, a caninized anti‑NGF monoclonal antibodies (ranevetmab) have been developed and shown to alleviates pain in dogs with degenerative joint disease [27]. Lokivetmab, a caninized anti-IL-31 monoclonal antibody, is another example of canine therapeutic antibodies, which reduces pruritus in dogs with atopic dermatitis [28]. Because the constant region is different among these therapeutic antibodies, e.g., canine IgG-D (c4G12), IgG-A (ranevetmab), and IgG-B (lokivetmab), the establishment of a mass production system for each canine IgG subclass is required to meet the demand for the application of therapeutic antibodies in the veterinary field. In this study, we confirmed that SpA from *S*. *aureus* has a low binding capacity for canine IgG-A, C, and IgG-D, as previously reported by Bergeron *et al*. [9]. In contrast, SpsQ, derived from a dog commensal CoPS (*S*. *pseudintermedius*), showed a higher binding capacity to canine IgG-A and IgG-D and improved the purification efficiency for those subclasses. The use of SpsQ as an affinity chromatography ligand may contribute to the establishment of a robust and efficient purification scheme for canine therapeutic antibodies whose IgG subclass is A or D.

Although we observed an improvement in the recovery rate of IgG-A and IgG-D by using r-SpsQ-immobilized resin, the rate was still lower than that of IgG-B. In this study, affinity chromatography was performed under a standard protocol for antibody purification, leaving room for improvement in the materials and conditions to optimize affinity chromatography for our purpose. For example, modification of the particle size of the resin is often employed in commercially available protein A resins. Because of its increased surface area and better ligand access, a resin of smaller particle size could lead to the improvement of binding capacity [29]. Multimerization of a certain Ig-BD is also applied to some commercialized protein A resins. Polymerized domain B of SpA is reported to have a high Ig-binding capacity, and the number of domain B used for polymerization positively correlated with the binding capacity, linearly in polymer size, up to octamers [30]. Moreover, the binding capacity of a resin can also be influenced by various conditions, such as the residence time, antibody concentration, and pH of the sample. Optimization of materials and techniques for affinity chromatography using SpsQ is necessary to further improve the purification efficiency of canine antibodies.

The limitation of this study is the lack of experiments using live *S*. *pseudintermedius* isolates. Although the stronger canine IgG binding of SpsQ could support our hypothesis that *S*. *pseudintermedius* has evolved with its host animal species and adapted to the dog immune system, the advantages of SpsQ for *S*. *pseudintermedius* in evading dog immune system are still speculative. Interestingly, we found five Ig-BDs in SpsQ of our *S*. *pseudintermedius* isolates in Japan, although a previous report identified SpsQ with four Ig-BDs in *S*. *pseudintermedius* ED99, which was isolated in Scotland [31,32]. The *spsQ* locus is reported to be a hot spot for recombination and gene transfer in *S*. *pseudintermedius*, and is involved in acquiring lineage-associated genes with adaptive functions [18]. Because the identical *spsQ* gene with five Ig-BDs was found all over the world, our *S*. *pseudintermedius* isolates might have undergone evolution the same as others. Functional consequences of the difference in Ig-BD repeats need to be assessed in experimental infection models or cell-based assays using live bacteria.

So far, seven CoPS species have been reported: *S*. *aureus*, *S*. *intermedius*, *S*. *schleiferi* subsp. coagulans, *S*. *hyicus*, *S*. *lutrae*, *S*. *delphini*, and *S*. *pseudintermedius* [33]. The major CoPS differs by animal species, and host specificity has been suggested for some CoPS. The major natural hosts of *S*. *intermedius*, besides *S*. *aureus* and *S*. *pseudintermedius*, are Carnivora (e.g., *Canis*, *Mustela*, *Vulpes*, *Procyon*), and the major hosts of *S*. *hyicus* are Artiodactyla (e.g., *Bos*, *Sus*) [34]. Although expression of protein A and its binding to IgGs were observed in *S*. *intermedius* and *S*. *hyicus* [35,36], the binding characteristics of their protein A have not been reported. Thus, further research is required to examine whether protein A of each CoPS can most efficiently bind to antibodies of their respective major host animal species, reflecting evolutionary adaptation to the host.

In conclusion, we identified the nucleotide sequence of SpsQ from *S*. *pseudintermedius* isolates, which contained predicted Ig-BDs. We observed that the binding capacity of r-SpsQ to canine IgG-A and IgG-D was higher than that of r-SpA, supporting, when used as affinity ligands, a higher recovery rate of IgG-A and IgG-D in affinity chromatography. Optimization of the chromatography protocols and further characterization of SpsQ as an affinity chromatography ligand would contribute to the establishment of a cost effective and practical purification process for canine therapeutic antibodies, paving the way for the development of antibody-based therapies for canine diseases.

## Supporting information

S1 Fig The original image of the SDS-PAGE gel. The original uncropped and unadjusted image of the SDS-PAGE gel, related to (a) Fig 2C and (b) Fig 4A.
(TIF)
