# Supplementary figures and images for: Characterization of SpsQ from Staphylococcus pseudintermedius as an affinity chromatography ligand for canine therapeutic antibodies
Source: PLoS One. 2023 Jan 26;18(1):e0281171. doi: 10.1371/journal.pone.0281171 (PMC9879442; doi:10.1371/journal.pone.0281171)

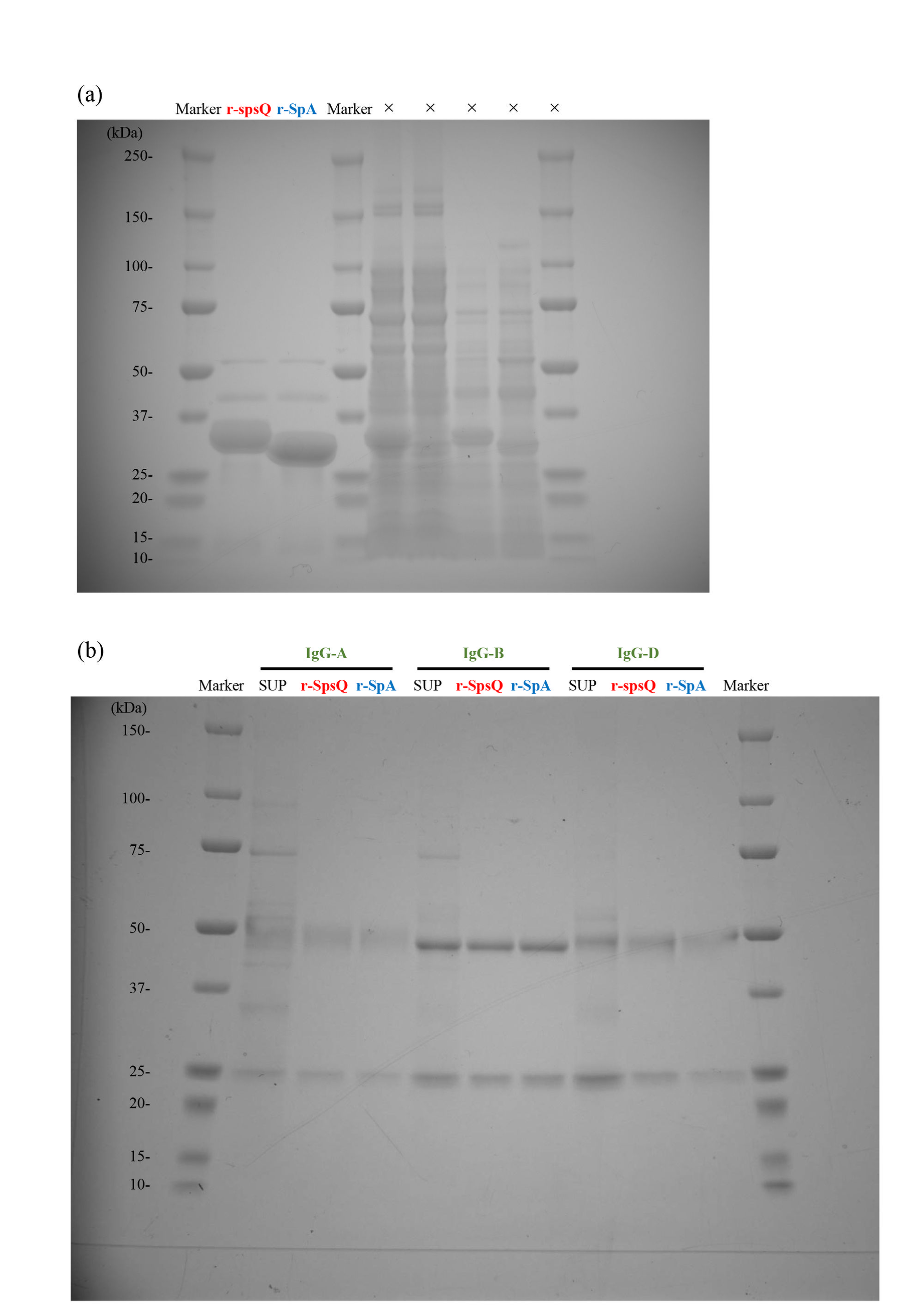

Supplement: S1 Fig — The original uncropped and unadjusted image of the SDS-PAGE gel, related to (a) Fig 2C and (b) Fig 4A. (TIF) [file pone.0281171.s001.tif]
